# Validation of a novel method to create temporal records of hormone concentrations from the claws of ringed and bearded seals

**DOI:** 10.1093/conphys/coaa073

**Published:** 2020-08-25

**Authors:** Shawna A Karpovich, Larissa A Horstmann, Lori K Polasek

**Affiliations:** 1 Alaska Department of Fish and Game, Marine Mammal Program, Fairbanks, AK 99701, USA; 2 College of Fisheries and Ocean Sciences, University of Alaska, Fairbanks, AK 99775, USA; 3 Alaska Department of Fish and Game, Marine Mammal Program, Juneau, AK 99811, USA

**Keywords:** Cortisol, keratin, phocid, pinniped, progesterone, steroid hormone

## Abstract

Ringed (*Pusa hispida*) and bearded seals (*Erignathus barbatus*) inhabit vast and often remote areas in the Arctic, making it difficult to obtain long-term physiological information concerning health and reproduction. These seals are experiencing climate-driven changes in their habitat that could result in physiological stress. Chronic physiological stress can lead to immunosuppression, decreased reproduction and decreased growth. Recently, keratin has become a popular matrix to measure steroid hormones, such as stress-related cortisol and reproduction-related progesterone. We developed and validated methods to extract cortisol and progesterone from the claws of adult female ringed (*n* = 20) and bearded (*n* = 3) seals using enzyme immunosorbent assays. As ringed and bearded seal claws grow, a pair of dark- and light-colored bands of keratin is deposited annually providing a guide for sampling. Two processing methods were evaluated, removal of claw material with a grinding bit or grinding followed by mechanical pulverization (102 paired samples from six claws, two each from three seals). Adding the mechanical pulverization step resulted in a 1.5-fold increase in hormone extraction. Progesterone from the proximal claw band was evaluated to biologically validate claw material as a measure of pregnancy in ringed seals (*n* = 14). Claws from pregnant seals had significantly higher claw progesterone concentrations than from non-pregnant seals. This suggests that the elevated progesterone associated with gestation was reflected in the claws, and that the most proximal claw band was indicative of pregnancy status at time of death. Thus, although the sample size was low and the collection dates unbalanced, this study demonstrates the potential to use claws to examine an extended time series (up to 12 yrs) of cortisol and progesterone concentrations in ringed and bearded seal claws.

## Introduction

Arctic regions are undergoing declines in sea ice duration, extent and thickness (Shaftel *et al.*, 2015) and increases in water temperature (Stroh *et al.*, 2015). Ringed (*Pusa hispida*) and bearded (*Erignathus barbatus*) seals inhabit the Arctic, and in Alaska are found in the Beaufort, Chukchi and Bering seas (Allen and Angliss, 2010, Burns, 1970). For Arctic phocids, suitable sea-ice habitat is essential for resting, foraging and pup rearing (Gjertz and Lydersen, 1983, Kelly *et al.*, 2010). Decreases in sea ice extent may lead to changes in the timing, quality and quantity of prey, increases in water and air temperature, severe weather and exposure to disturbances, pathogens and toxins (e.g. Burek *et al.*, 2008). These changes could have considerable influence on the diet, health and reproduction of ringed and bearded seals, making them especially vulnerable to current and future habitat alterations. Therefore, an examination of how habitat changes influence the health, reproduction and survival of ice-dependent seals is warranted. Ringed seal reproductive rates are often estimated by the proportion of pups harvested by subsistence hunters (Crawford *et al.*, 2015, Ferguson *et al.*, 2005, Harwood *et al.*, 2000, Stirling, 2005). However, many factors (e.g. ice conditions, hunter preference, seasonal distribution) may influence the harvest proportions and bias these estimates. Reproduction can also be assessed by counting pups, but pupping locations are spread over large areas of ice (Harwood and Stirling, 1992) and can occur in snow covered dens (Born *et al.*, 2004) making pups difficult to detect. Alternatively, reproduction can be evaluated by examining reproductive tracts of harvested animals (Crawford *et al.*, 2015, Holst *et al.*, 1999), but this method is most accurate on samples collected in the autumn and winter when harvests are less common and assessing reproduction from tracts collected in other seasons can overestimate the number of successful births (Crawford *et al.*, 2015). Therefore, developing a method using other tissues to estimate pregnancy may reduce some of the difficulties associated with estimating pregnancy rates in these species.

If the habitat changes that ringed and bearded seals are experiencing are perceived as stressors, the hypothalamic-pituitary-adrenal axis will be activated leading to a complex suite of physiological and behavioral responses, including the release of glucocorticoids (Reeder and Kramer, 2005). Glucocorticoids, such as cortisol, then mobilize body energy stores to allow animals to cope with change or flee from danger (e.g. Romero and Butler, 2007). Thus, concentrations of cortisol are commonly used as an index of physiological stress in mammals (Gulland *et al.*, 1999, Möstl and Palme, 2002, Ortiz and Worthy, 2000, Thomson and Geraci, 1986). Short-term stress (triggering the fight-or-flight response) is an adaptive mechanism that increases the likelihood of survival; however, chronically elevated cortisol can result in negative physiological effects, such as immunosuppression, decreased reproduction (Dobson and Smith, 2000) and decreased growth (e.g. Möstl and Palme, 2002). Another hormone, progesterone, has similarly been used to assess reproduction in pinnipeds (Boyd, 1991, Greig *et al.*, 2007). Therefore, evaluating the levels of cortisol and progesterone would be especially valuable considering the effects of climate change on the Arctic, which could result in changes in chronic physiological stress or reproductive success for ice-dependent seals.

Long-established matrices to measure steroid hormones in pinnipeds include blood, feces, urine, blubber and saliva (Atkinson, 1997, Atkinson *et al.*, 2015). The period represented by these sample types is often short or difficult to estimate making it problematic to interpret the hormones measured. These samples are generally difficult and costly to collect in free-ranging wildlife. For most seals, they are almost impossible to obtain from the same individuals over time, which precludes long-term assessment of hormones. Progesterone concentrations can be difficult to interpret from single samples representing short periods, because the pinniped reproductive cycle includes a several month delayed implantation of the fertilized cells, during which circulating progesterone concentrations are similar between pregnant and non-pregnant animals (Greig *et al.*, 2007, Guinet *et al.*, 1998, McKenzie *et al.*, 2005). Cortisol concentrations from the above-mentioned tissues could also be difficult to interpret as capture or harvest events can cause acutely elevated levels of cortisol to be released into circulation that may obscure the pre-disturbance levels (Gulland *et al.*, 1999, Ortiz and Worthy, 2000, Thomson and Geraci, 1986). Challenges assessing physiological stress and reproduction over time highlight the need to develop methods for novel tissues, especially for species that are difficult to sample, like ringed and bearded seals.

Recently, the assessment of steroid hormones in hair as biomarkers for reproduction or chronic physiological stress has gained popularity (Koren *et al.*, 2019). Hair cortisol concentrations have been correlated with climate-related changes in habitat and contaminant load in polar bears (*Ursus maritimus*; Bechshøft et al., 2012a,b, 2013, 2015), nutritional and social stress in grizzly bears (*Ursus arctos*; Bryan *et al.*, 2013, Macbeth *et al.*, 2010) and hunting pressure in wolves (*Canis lupus*; Bryan *et al.*, 2015). However, mammalian hair has a relatively short period of growth (~1.5 mo in phocids; Ashwell-Erickson *et al.*, 1986). Thus, sampling keratinous tissue with an extended growing period would be more useful to examine long-term trends in hormone concentrations.

Seal claws grow continuously, and stable isotopes stored in ice seal claws contain diet information spanning up to the previous 12 years (Boucher *et al.*, 2020, Carroll *et al.*, 2013, Ferreira *et al.*, 2011). Whale baleen is also a continuously growing keratinous tissue that contains long-term records of several steroid hormones (Hunt *et al.*, 2014, 2017) and baleen progesterone concentrations have been correlated with known pregnant and non-pregnant periods (Hunt *et al.*, 2016). Similarly, steroid hormones have been extracted from the hooves of cattle (Comin *et al.*, 2014) and the claws of turtles (Baxter-Gilbert *et al.*, 2014), dogs (Fusi *et al.*, 2018, Veronesi *et al.*, 2015) and chameleons (Matas *et al.*, 2016), yet no such studies have examined marine mammal claws. Therefore, ice seal claws that grow continuously and have visibly discernable annual banding (Benjaminsen, 1973, McLaren, 1958) are an ideal matrix to examine long-term trends in hormone concentrations. This may be especially useful as ice seal habitats undergo climate change-related alterations.

**Table 1:** Individual IDs, collection date, pregnancy status, tooth and claw derived ages and proximal band progesterone concentration from adult female ringed seal claws. Pregnancy status was determined by examination of written collector notes or visual confirmation of the reproductive tract. Tooth ages were estimated using cementum growth layers and claw ages are the number of bands counted on the claw followed by a + indicating the estimated minimum age, as the number of bands lost to wear is unknown. ‘Prox. Claw Prog.’ is the progesterone measured in the most proximal claw band that was examined for correlation with the pregnancy status at the time of death

Individual ID	Collection date	Pregnancy status	Tooth age	Claw age	Prox. claw prog. (pg/mg)
ADFG:11SH015	2-Oct-11	Non-pregnant	12	11+	79.88
ADFG:11SH016	2-Oct-11	Non-pregnant	13	8+	78.82
ADFG:11SH099	13-Oct-11	Non-pregnant	18	9+	82.20
ADFG:10SH005	15-Oct-10	Non-pregnant	24	8+	103.68
ADFG:11GAM003	13-Nov-11	Non-pregnant	10	11+	44.41
UAM:Mamm:36830	12-Jan-67	Pregnant		11+	123.06
UAM:Mamm:36825	28-Jan-63	Pregnant		9+	111.73
UAM:Mamm:122131	10-Feb-67	Pregnant		11+	212.86
UAM:Mamm:36826	10-Feb-64	Pregnant		9+	118.35
ADFG:10SH053	10-Nov-10	Pregnant	25	12+	65.68
ADFG:10SH061	10-Nov-10	Pregnant	9	8+	93.52
UAM:Mamm:19062	13-Nov-66	Pregnant		6+	155.72
ADFG:14SH018	15-Nov-14	Pregnant	20	9+	155.15
UAM:Mamm:19059	30-Nov-65	Pregnant		8+	110.76

Within this study, we developed and validated a technique for monitoring chronic physiological stress and reproductive status for ringed and bearded seals. The four main goals of our study were to (1) develop a protocol for hormone extraction from ringed and bearded seal claws, (2) validate enzyme immunosorbent assays (EIAs) for cortisol and progesterone using extracts from ringed and bearded seal claws, (3) compare methods for collecting claw powder by grinding with a diamond-tipped bit with and without an added mixer-mill pulverization step and (4) compare progesterone concentrations from the most recently deposited claw material (proximal band) to pregnancy status at time of harvest (pregnant *vs.* non-pregnant).

## Methods

### Sample collection

Claws were collected from 20 adult female ringed (3 laboratory validations, 3 processing methods, and 14 biological validations) and 3 adult female bearded seals (laboratory validations) harvested by Alaska Native subsistence communities in the Chukchi and Bering seas, Alaska, and were obtained from the University of Alaska Museum Mammalogy Collection (UAM:Mamm), the Alaska Department of Fish and Game (ADF&G) and the North Slope Borough (NSB). Laboratory validations required large samples, so extracts from two to four claws per seal were pooled from three ringed seals (NSB:RS602, NSB:RS51800 and NSB:RS700) and three bearded seals (NSB:BS602, NSB:BS702 and NSB:BS080801), respectively. Pools were created so that each individual seal was represented equally. To compare processing methods, the two longest claws each from three ringed seals ADFG:11GAM009, ADFG:10SH005 and NSB:2014–01 were used. Lastly, individual progesterone concentrations from the most proximal claw band from 14 adult female ringed seals were evaluated to compare pregnancy status at the time of death to claw progesterone concentrations (Table 1). Claws from the 1960s were stored at UAM in paper envelopes, claws collected from 2010 to 2014 were stored frozen as whole flippers, then removed from the flesh and stored in paper envelopes ≤1 yr prior to processing.

A fetus was collected with three of the six ringed seals from the museum collection (UAM:Mamm:19062, UAM:Mamm:19059 and UAM:Mamm:122131; Table 1), otherwise pregnancy status was determined by museum notes acquired from the original collectors. ADF&G collected claws and reproductive tracts from eight harvested ringed seals (Table 1) and pregnancy was determined by presence or absence of uterine implant sites as outlined in Crawford *et al.* (2015).

**Figure 1:**
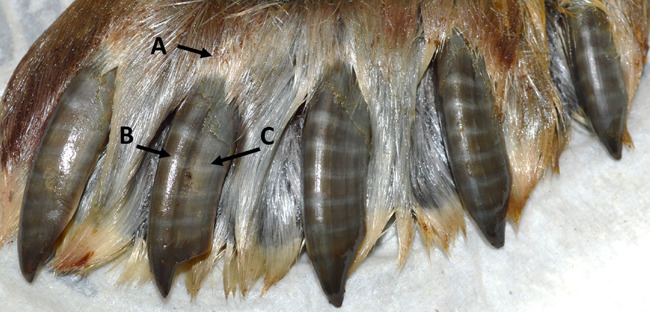
Front flipper from a subsistence harvested ringed seal showing the distinct claw bands. Pairs of bands are deposited annually (Benjaminsen, 1973, McLaren, 1958). The most proximal band (A), used for biological validations in this study, is hidden under the skin and fur at the insertion point of the claw. The central ridge (B) is the thickened dorsal and central portion of the claw (not sampled) and the lateral walls (C) are the area of the claw sheaths sampled in this study.

Seal age was estimated using teeth or claws (Benjaminsen, 1973, McLaren, 1958). For a subset of seals, a lower canine was sent to Matson’s Laboratory, Milltown, Montana, USA, and ages were estimated by counting annual growth layers (cementum rings) in the tooth. Also, age was estimated using the banding pattern on the claws (Fig. 1) (Benjaminsen, 1973, McLaren, 1958). Because claw bands are lost from the distal end of the claw during wear, claw ages represent a minimum age estimate, denoted by a ‘+’ after the estimate (Table 1).

### Claw processing

Claws were submerged in a room temperature water bath until the keratinous claw sheath could be removed from the underlying bone and tissue (~1–14 days), then stored in paper envelopes at room temperature until processed. At the time of processing, claws were again soaked for 1–4 days to soften any remaining tissues adhered to the claw (see cuticle-like tissues at the top of the claws in Fig. 1), which were removed with a small weighing spatula. After soaking in the water baths, the claws were often coated with contaminants associated with other tissues in the flipper (e.g. blood and oils). To remove surface contaminants, claws were sonicated in deionized (DI) water for 30 min and immersed in 2:1 chloroform:methanol for 30–60 s. Finally, the outer surface was wiped with a cotton swab, and the inner surfaces scrubbed with a straw-cleaning brush, both wetted with 2:1 chloroform:methanol. Immersion and scrubbing/wiping was repeated four times, or more if the cotton swab continued to show discoloration from water bath contamination. Claws were dried in open containers at room temperature for ≥24 h prior to grinding.

Powdered samples of claw material were collected using a Dremel® tool at ~17 000 rpm with a diamond-coated engraving tip (part 7134; 2 mm bit diameter). For validations and comparison between processing methods, serial samples were collected from each claw. Sampling was restricted to the lateral walls of the claw sheaths, because the dorsal ridge of the claw contains keratin that is continuously deposited from the underlying tissue, which would confound the estimation of the timing of deposit (Ethier *et al.*, 2010). During sampling, the claw was ground to ~2 mm in depth and followed the contour of the visible bands. The claw powder was collected in a tin weighing dish, ~3–5 ml of DI water was added, mixed into a slurry, then poured into a 5-ml glass vial. Open vials were dried at ≤60°C for ≤6 days. To avoid cross contamination, gloves and weighing dishes were changed and the work area, claw and Dremel® were cleared with forced air between each sample.

### Sample powdering methods

Bearded seal claws were processed first, as they are large (~6.5 × 1.4 cm) and collecting consistent powder using the grinding tip was unproblematic. Ringed seal claws are smaller (~5 × 0.8 cm) and large chunks of claw material often broke off while grinding, making the powder consistency more variable. Therefore, different processing methods were tested on pairs of claws from three individual ringed seals. One claw was processed by grinding with the Dremel® or grinding followed by pulverization of the powder using a Retsch® Mixer Mill MM400. Samples were collected from multiple bands along each claw (51 paired samples, *n* = 102). Post-grinding claw material was transferred into 2-ml Sarstedt® screwcap micro-centrifuge tubes with a silicon gasket, and for the grind+mill method, two 5-mm steel ball bearings were added to each tube and the sample was pulverized into a finer powder at 30 Hz for 15 min in the mill.

### Hormone extraction

For hormone analyses, 5 ± 0.5 mg of dried claw powder was weighed to the nearest 0.1 mg and extracted in 1 ml of 100% ACS grade methanol by slowly rotating for 24 h. All samples were centrifuged for 13 min at 10°C and 10 500 g. Supernatant was transferred to a new 2-ml tube, and pellets were rinsed with 0.2 ml of methanol, centrifuged (same as above), and the rinse supernatant was added to the sample’s extract. If less than 5 mg of claw powder was collected, the amount of methanol used during extraction and rinsing was reduced, so the ratio of powder to methanol was consistent among samples. The methanol extracts were frozen at ≤−20°C until analyzed (~0–3 months).

### Validations

Arbor Assays® kits (Ann Arbor, MI, USA) for progesterone (catalog # K025) and cortisol (catalog # K003) were validated using parallelism and accuracy tests. For validations, extracts from multiple claw bands and individuals were pooled. To verify that the claw extracts did not affect the kit hormone detection capabilities across the range of detection concentrations, accuracy tests were conducted. Kit standards were serially diluted, then combined with equal parts of the pooled sample. Accuracy was determined by plotting the expected and observed hormone concentrations of the standard-pool mixtures in SigmaPlot 13. To verify that the kit could accurately measure hormones from the claw extracts within the detection range, parallelism tests were conducted. Pooled claw extracts were serially diluted and assayed. The percent binding of extracts and standards was plotted against hormone concentration (pg/ml) expressed on a log scale.

### Progesterone and cortisol hormone assays

Tubes of sample supernatant were removed from the freezer and centrifuged (13 min, 10 °C, 10500 g) to remove any residual powder from solution. The supernatant was placed into glass culture tubes and dried under forced air. For method comparisons and biological validations, 0.175 ml of ringed seal claw extract was dried and reconstituted with 0.12 ml of buffer, then analyzed for progesterone concentrations. Samples, standards, controls, non-specific binding and blank wells were assayed in duplicate. Results are presented as picograms of hormone per milligram of claw (pg/mg).

### Biological validation

Based on the manner that claws grow, the most proximal claw material (base) contains the most recently deposited keratin. Therefore, for ringed seals (*n* = 14), claw progesterone concentrations from the most proximal material were compared to pregnancy status at the time of death (Table 1).

### Statistics

All samples were run in duplicate and consistency between duplicates was examined using the mean intra-assay % coefficient of variation (*CV*). Plate-to-plate consistency was examined by calculating inter-assay % *CV* from the mean values of high and low controls included on each plate. For accuracy tests, the slope and 95% confidence interval *(CI)* of each line were examined for the inclusion of 1 (slope of a 1 to 1 relationship). Testing for parallelism was conducted with an analysis of covariance wherein two different models were fit and compared. One model was parameterized with parallel lines fitting paired sample and standard data while another model allowed for the slopes to vary between the sample and standards. A likelihood ratio test was subsequently performed to assess which of these two models were most supported by the data and is reported as ‘*F* statistic, *P* value’. To account for unequal variance, the comparison between the grind-only and grind+mill processing methods was conducted using a Wilcoxon Signed Ranks test, values are reported as ‘median value (sample size, range)’*,* and test results as *‘Z* statistic*, P* value’. For biological validations, predicted claw progesterone concentrations were compared using a Mann–Whitney sum test and values are reported as ‘median value (sample size, range)’ and test results as *‘U* statistic, *P* value’. All tests were considered significant at *P* ≤ 0.05.

## Results

### Validations

**Figure 2:**
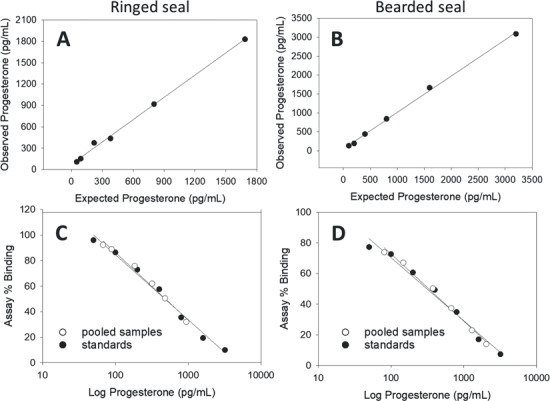
Validations of progesterone EIAs using pooled methanol extracts from powdered ringed (A, C) and bearded seal (B, D) claws. Accuracy tests (A, B) were conducted by combining equal parts of pooled sample with each standard and the *CI* of each slope included 1 (slope of a 1 to 1 correlation). Parallelism tests (C, D) compared the slope of serially diluted pooled samples with serially diluted standards; the *CI*s of the slopes had strong agreement. Slopes, 95% *CI* of slopes and *R^2^* values are reported in Table 2.

**Figure 3:**
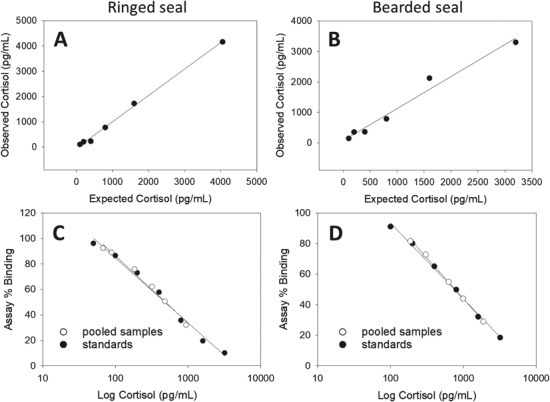
Validations of cortisol EIAs using pooled methanol extracts from powdered ringed (A, C) and bearded (B, D) seal claws. Accuracy tests (A, B) were conducted by combining equal parts of pooled sample with each standard and the *CI* of each slope included 1 (slope of a 1 to 1 correlation). Parallelism tests (C, D) compared the slope of serially diluted pooled samples with serially diluted standards; the *CI*s of the slopes had strong agreement. Slopes, 95% *CI* of slopes and *R^2^* values are reported in Table 2.

Regression lines created by plotting the expected and observed concentrations from pooled claw extract/standard combinations (accuracy tests) were linear (Figs 2A, 2B, 3A, 3B). The 95% *CI* for all accuracy tests included 1 and had an *R^2^* ≥ 0.976 (Table 2). This indicates that both high and low levels of progesterone and cortisol could be accurately differentiated using extracts from ringed and bearded seal claw powder. Pairs of regression lines created by plotting the percent binding and the hormone concentration of serially diluted sample and standard pools (parallelism tests, Figs 2C, 2D, 3C, 3D), had similar slopes and showed substantial overlap of the 95% *CI*s between sample/standard pairs with *R^2^* ≥ 0.983 (Table 2). The likelihood ratio test rejected the model where slopes could vary, thus confirming that the serially diluted sample lines were parallel to the corresponding serially diluted standard lines (*F* = 0.108, *P* = 0.744). This indicates that no significant interference or magnification of binding was observed while using extracts from claw powder in the EIAs.

**Table 2:** Slope, upper and lower 95% *CI*, *R^2^* and the corresponding figure panel for each line created during validations of progesterone and cortisol EIAs using pooled methanol extracts from powdered ringed and bearded seal claw material.

Species	Hormone	Test	Slope	Upper 95% *CI*	Lower 95% *CI*	*R^2^*	Figure
Ringed	Progesterone	Accuracy	1.04	1.12	0.96	0.994	2A
Ringed	Progesterone	Parallelism (standards)	−22.12	−19.41	−24.83	0.989	2C
Ringed	Progesterone	Parallelism (samples)	−22.96	−19.32	−26.60	0.987	2C
Bearded	Progesterone	Accuracy	0.96	1.01	0.91	0.998	2B
Bearded	Progesterone	Parallelism (standards)	−17.87	−14.93	−20.81	0.983	2D
Bearded	Progesterone	Parallelism (samples)	−19.03	−16.79	−21.27	0.992	2D
Ringed	Cortisol	Accuracy	1.04	1.12	0.97	0.999	3A
Ringed	Cortisol	Parallelism (standards)	−21.57	−18.61	−24.53	0.990	3C
Ringed	Cortisol	Parallelism (samples)	−21.62	−18.38	−24.86	0.993	3C
Bearded	Cortisol	Accuracy	1.05	1.28	0.82	0.976	3B
Bearded	Cortisol	Parallelism (standards)	−21.53	−19.69	−23.36	0.996	3D
Bearded	Cortisol	Parallelism (samples)	−23.17	−21.44	−24.91	0.998	3D

Intra-assay % *CV*s for individual samples run in duplicate averaged 6.5% for cortisol (*n* = 126) and 4.5% for progesterone (*n* = 126). Inter-assay % *CV*s derived from high and low controls included on each plate were 11.2% (*n* = 5) and 7.4% (*n* = 12), for cortisol and progesterone, respectively.

### Sample powdering methods

**Figure 4:**
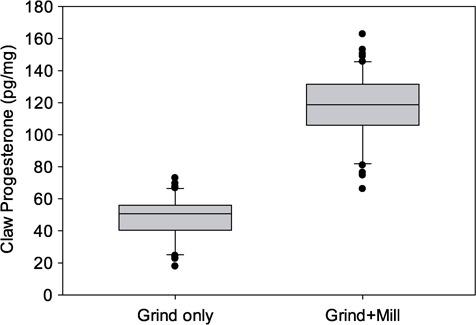
Effect of claw processing on the progesterone concentrations from claw bands of three adult ringed seals. Paired claws from each individual seal were processed using two methods. The bands of one claw were processed using the ‘Grind only’ method (*n* = 51) and bands from a second claw were processed using the ‘Grind+Mill’ method (*n* = 51). Progesterone concentrations were significantly higher when the mill pulverization step was added. Boxplots encompass the 25% and 75% quartiles, and the median is depicted by a line in the middle of the gray box. Whiskers extend to the 10th and 90th percentiles, and values beyond that (outliers) are shown as individual points.

Adding the mill pulverizing step significantly increased the concentration of progesterone extracted from the claw material (Fig. 4). Concentrations of claw progesterone extracted from the grind-only powder was 50.6 pg/mg (*n* = 51, 17.8–72.9 pg/mg), which was significantly lower than concentrations from the grind+mill powder of 118.7 pg/mg (*n* = 51, 66.1–162.8 pg/mg) (*Z* = 6.2, *P* < 0.001). Subsequently, validations for ringed seal samples (Figs 2 and 3) and all individual ringed seal claw values (Table 1 and Fig. 4) were processed using the grind+mill method.

### Biological validation

**Figure 5:**
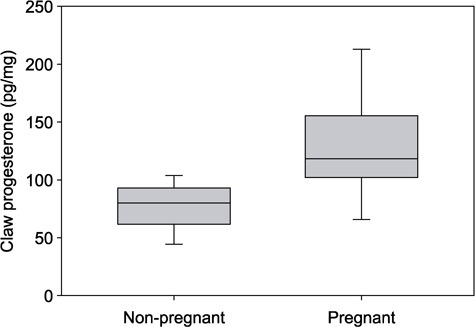
Progesterone concentrations in the proximal band of ringed seal claws extracted using the ‘Grind+Mill’ method. Seals were identified as either non-pregnant (*n* = 5) or pregnant (*n* = 9) at time of death based on observation of a fetus or an embryonic implant site. Claw progesterone concentrations were significantly higher comparing seals that were pregnant at time of death to seals that were not. Boxplots encompass the 25% and 75% quartiles, the median value is depicted by a solid line in the middle of the gray boxes, and whiskers extend to the 10th and 90th percentiles. Outliers were not identified due to the small sample sizes.

Progesterone concentrations in the most recently deposited claw material from adult female ringed seals that were identified as pregnant at the time of death was 118.3 pg/mg (*n* = 9, 65.7–212.9 pg/mg), which was significantly higher than claw progesterone concentrations from seals that were identified as non-pregnant 79.9 pg/mg (*n* = 5, 44.4–103.7 pg/mg) (*U* = 5, *P* = 0.02), (Table 1 and Fig. 5). However, when claw progesterone values from January and February harvested pregnant seals were removed and comparisons were only made among seals harvested in October and November, the difference was no longer significant (*U* = 5, *P* = 0.151).

## Discussion

Recent studies document that keratinized tissue can store long-term information associated with physiological stress and reproduction. Cortisol or corticosterone in the claws of other species have been correlated with proximity to roads (Baxter-Gilbert *et al.*, 2014), premature birth (Veronesi *et al.*, 2015) and social dominance associated with body size (Matas *et al.*, 2016). Progesterone concentrations from other keratinized tissues, such as hooves (Comin *et al.*, 2014) and baleen (Hunt *et al.*, 2016), have been correlated with known pregnancies. The validations presented here show that both cortisol and progesterone can be extracted from ringed and bearded seal claws, and to our knowledge, this is the first study to measure hormone concentrations in pinniped claws. This may be especially important for these ice-dependent seals that are vulnerable to climate change-related changes such as declines in duration, quality, or quantity of sea ice.

The visibly discernable annual claw banding in ice seal claws (Benjaminsen, 1973, McLaren, 1958) makes the collection of several years of information from one sampling event attractive. However, several overlapping longitudinal layers of keratin were observed in bearded seal claws (Benjaminsen, 1973). If this is the case in the present study, the arrangement of these layers would mean that the 2 mm depth sampled would contain two or three distinct layers. However, the previous report does not describe which portion of claw was cross sectioned and due to the width of the claw material shown (~4 mm; Benjaminsen, 1973), we suspect the thickened central ridge of the claws was used. Examinations of other mammalian claws have concluded that keratin is continuously deposited under the central ridge, while the lateral walls are deposited exclusively at the insertion point of the claw (germinal matrix; Ethier *et al.*, 2010). In other words, time series information stored in the central ridge of claws is confounded by layering of keratin over time, while the lateral walls contain an undiluted time series of information. In this study, we sampled exclusively from the lateral walls, and only sampled the most proximal band where, even if additional material were to be deposited under the lateral walls of the claws, the animal was harvested before it would have been possible.

The comparison of progesterone from paired claws collected from individual ringed seals requires that the hormone concentrations are similar among neighboring claws. Carroll *et al.* (2013) found that stable isotope signatures did not vary among claws sampled from individual ringed seals; therefore, we assumed that hormone deposits would also be similar. Yet, when the mill pulverization step was added to a powder collected from a second claw from the same seal, the concentration of progesterone extracted was 1.5 times higher. This suggests that methods used during claw processing can have a large influence on the hormone concentrations reported. Several methods have been utilized to process claws from other species for steroid hormone extraction, including mincing of claw tips (Fusi *et al.*, 2018), collecting shavings of claw tips (Veronesi *et al.*, 2015), freezing and crushing claw tips (Baxter-Gilbert *et al.*, 2014) and producing powder by grinding or grinding then pulverizing in a mill (this study). Pulverization in a mill is preferable to other claw processing methods, because it is mechanical and timed, which decreases the likelihood of human error adding variability to the sample consistency. Mill grinding was also preferred for preparation of hair samples for hormone extraction (e.g. Meyer *et al.*, 2014). Additionally, the mill pulverization step resulted in higher concentrations of hormones extracted, allowing for smaller samples sizes or tissues with lower hormone concentrations to be analyzed. Regardless of the method used, with consistent processing steps, the hormone concentrations measured within each study should be comparable. However, direct comparisons of hormone concentrations among studies that use dissimilar processing methods may be invalid.

In phocids, serum progesterone can be used to detect pregnancy (Gardiner *et al.*, 1996, Reijnders, 1990). In the present study, ringed seals that were pregnant when the claw was collected had higher progesterone concentrations in the proximal claw material than those that were non-pregnant. This suggests that the progesterone deposited into the claw was correlated with circulating progesterone and that the proximal band of claws indicated reproductive status during the period of keratin deposition. A similar comparison of progesterone concentrations in bearded seal claws could not be conducted because all harvested females with information about reproductive status were either pregnant or post-partum and claws from non-pregnant individuals were unavailable for comparison. Further research is required to determine if claw progesterone can also be used as an indicator of pregnancy in bearded seals. Yet, the ringed seal results suggest that retrospective examinations of reproduction from the previous 6 to 12 years could be conducted by analyzing serial bands along the length of the claws.

During phocid gestation, serum progesterone levels rise gradually (Boyd, 1991, Reijnders, 1990). This implies that pregnancy would be easier to detect closer to pupping (May–June for ringed seals; Kelly *et al.*, 2010). In this study, the harvest dates were not balanced, and for some pregnant seals the proximal bands contained more material deposited while the seal was pregnant including some material deposited during late pregnancy when progesterone was highest. This may explain why the difference between pregnant and non-pregnant claw progesterone levels became non-significant after the removal of seals that had been gestating for 3 additional months. Accordingly, future studies with larger sample sizes and balanced harvest dates would be beneficial. However, unbalanced harvest dates would not affect all other bands along the claws as those bands were deposited during previous years and thus contain keratin deposited during the entire gestation period.

This study only presents ringed seal claw progesterone concentrations from the most proximal claw band. However, the validations and methods presented here may also be used to assess cortisol and progesterone from other bands along the length of ringed and bearded seal claws. As some claws contained up to 12 years’ worth of bands, this study introduces the potential to describe changes in progesterone and cortisol for a substantial portion of a seal’s lifetime. Assuming the lateral walls of seal claws contain an undiluted time series of information, the hormones in claws represent concentrations in circulation and are inert once deposited; analyses of serial bands of claw growth give researchers the ability to examine annual pregnancy status and changes in chronic physiological stress, among other things. This would be especially valuable for retrospective studies examining responses to unexpected changes when researchers do not have notice to collect pre-event data (e.g. unusual mortality events, extreme weather, chemical spills, or any other abrupt changes). Furthermore, the long-term physiological data available from claws could contribute to species management and conservation planning and be used for a broad range of ecological studies.

### Recommendations for future studies

(1) Standardized processing methods should include a mixer-mill pulverization step so that claw hormone concentrations can be comparable among studies. (2) Studies examining the timing and pattern of keratin deposition into ringed and bearded seal claws would be invaluable, especially to confirm that the lateral walls of the claw sheaths contain an undiluted time series of information. (3) In this study, claws were soaked in water for extended periods to allow the connective tissue that holds the claw sheath to the underlying bone to degrade. This required that the claw remain wet, otherwise the underlying tissues would dry, and the claw sheath would not disassociate. However, cortisol concentrations in primate hair decreased significantly with repeated exposure to soap and water (Hamel *et al.*, 2011, Li *et al.*, 2012). This raises the possibility that some claw hormone could have been lost in the water baths. We could not directly measure this because the baths contained oils and other substances from non-keratin tissues. Perhaps future studies using claws could prevent drying using alternate measures such as sealed containers or exposure to humidity. (4) The use of hormone concentrations stored in claws to recreate a timeline of hormone concentrations requires that claw hormones represent concentrations in circulation and remain inert once deposited. However, cortisol concentrations in baleen plates of bowhead whales (*Balaena mysticetus*; Hunt *et al.*, 2014) were highest at the base and declined moving toward the tip. This pattern suggests hormone loss over time or inclusion of hormone from sources other than circulation at the base of the baleen. Yet, the same pattern was not found in the concentrations of progesterone, another steroid hormone, in the baleen of North Atlantic right whales (*Eubalaena glacialis*; Hunt *et al.*, 2016). This demonstrates the need for a better understanding of the source and stability of steroid hormones in keratinous material.

## Funding

This work was supported by the National Marine Fisheries Service (grant number NA16NMF4390029).
